# A Nutrient-Based Cellular Model to Characterize Acetylation-Dependent Protein-Protein Interactions

**DOI:** 10.3389/fmolb.2022.831758

**Published:** 2022-03-23

**Authors:** Jérémy Loehr, Pata-Eting Kougnassoukou Tchara, Kevin Gonthier, Chahinez Noufi, Naomie Linteau, Étienne Audet-Walsh, Jean-Philippe Lambert

**Affiliations:** ^1^ Department of Molecular Medicine and Cancer Research Center, Université Laval, Quebec, QC, Canada; ^2^ CHU de Québec Research Center, Quebec, QC, Canada; ^3^ Big Data Research Center, Université Laval, Quebec, QC, Canada

**Keywords:** bromodomain, lysine acetylation, chromatin, ATP citrate lyase, interactome mapping, acetyl-CoA, acetate, functional proteomics

## Abstract

Cellular homeostasis requires the orderly expression of thousands of transcripts. Gene expression is regulated by numerous proteins that recognize post-translational modifications—in particular, the acetylation of lysine residues (Kac) on histones. In addition to affecting the general condensation state of the chromatin, acetylated histones act as anchor points for bromodomain (BRD)-containing adapter proteins. BRDs are the primary Kac reader domains in humans, and proteins containing them act as chromatin scaffolds that organize large networks of interactions to regulate transcription. To characterize BRD-dependent interaction networks, we established cell lines in which histone acetylation is dependent on acetate supplementation. To do this, we used genome editing to knock out ATP citrate lyase (ACLY), the enzyme responsible for converting citrate to oxaloacetate and acetyl-CoA in the cytoplasm and nucleus. In our cellular model, removing acetate from the culture medium resulted in the rapid catabolism of acetylated histones to restore the nucleocytoplasmic acetyl-CoA pool. Here we report the use of our new model in functional proteomics studies to characterize BRD-dependent interaction networks on the chromatin.

## 1 Introduction

Effective transcriptional regulation requires an array of post-translational modifications (PTMs) in mammalian cells, notably lysine acetylation (Kac). At its most fundamental level, Kac neutralizes the positive charge on the epsilon amine of the lysine sidechain. This reduces the compaction of nucleosomes, facilitating the transcription of the genetic material they contain. Beyond this effect, Kac also acts as a recruitment signal for bromodomains (BRDs), their main reader modules. BRD-containing proteins are evolutionarily conserved, and 42 are expressed in humans (Filippakopoulos et al., 2012). BRD-containing proteins are principally located in the nucleus (Gong et al., 2015) and contribute to numerous facets of transcriptional regulation (Fujisawa and Filippakopoulos, 2017). Dysregulation of their functions causes numerous diseases and conditions, supporting their therapeutic targeting (Filippakopoulos and Knapp, 2014). The hydrophobic nature of BRDs makes them well suited for chemical inhibition by small molecules, with approximately half the human BRDs predicted to be druggable (Vidler et al., 2012). Numerous BRD inhibitors are currently being evaluated in clinical trials; therefore, effective tools to delineate BRD functions are increasingly needed to help define the cellular contexts warranting their inhibition.

**FIGURE 1:**
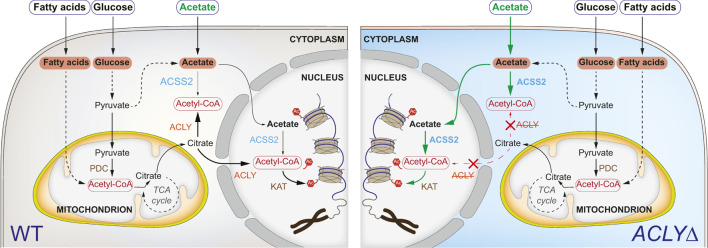
Acetyl-CoA metabolism and its role in Kac signaling. Simplified metabolic pathways contributing to Kac generation (left). In the absence of ACLY (right), the ACSS2 pathway can overcome the need for ACLY if sufficient acetate is available. Abbreviations: ATP-citrate synthase (ACLY), acetyl-coenzyme A synthetase (ACSS2), lysine acetyltransferase (KAT), pyruvate dehydrogenase complex (PDC).

The metabolite acetyl-coenzyme A (acetyl-CoA) is a necessary co-factor of acetyltransferases (KATs), making it a limiting factor for Kac production (Wellen et al., 2009). Two distinct acetyl-CoA pools exist in eukaryotic cells, namely the mitochondrial and nucleocytoplasmic pools (Figure 1). In the mitochondria, acetyl-CoA can be produced from pyruvate by the pyruvate dehydrogenase complex. Fatty acid oxidation and amino acid (e.g., leucine, isoleucine, and tryptophan) degradation can also generate acetyl-CoA. Lastly, acetyl-CoA synthetase short chain family member 1 (ACSS1) can catalyze the production of acetyl-CoA from acetate. Notably, mitochondrial acetyl-CoA metabolites do not contribute to epigenetic signaling since they cannot translocate to the cytoplasm. In human cells, a major source of the nucleocytoplasmic pool of acetyl-CoA is glycolysis, producing pyruvate that is subsequently used to generate citrate in the mitochondria through the tricarboxylic acid cycle. Citrate is exported out of the mitochondria via the dicarboxylate antiporter solute carrier family 25 member (SLC25A1) and is converted to acetyl-CoA and oxaloacetate in the cytoplasm and nucleus by the enzyme ATP citrate lyase (ACLY). Alternatively, non-mitochondrial acetate can be converted to acetyl-CoA by ACSS2 (Figure 1).

Sustained Kac signaling is necessary to maintain cellular homeostasis and survival. Systematic genome-wide CRISPR/Cas9 loss-of-function screens have revealed that *ACLY* is a commonly essential gene under normal laboratory growth conditions (e.g., depmap.org; (Tsherniak et al., 2017). The use of culture medium representative of adult human plasma (containing 60 additional polar metabolites and salt ions, including 40 μM acetate) allowed for the survival of *ACLY* knockout (KO; *ACLYΔ*) K562 cells (Rossiter et al., 2021). More specifically, Houston et al. reported that the essentiality of *ACLY* can be overcome through acetate supplementation of traditional culture medium, enabling acetyl-CoA production via the ACSS2 pathway (Houston et al., 2020) (Figure 1). Secession of acetate supplementation in *ACLYΔ* HT1080 cells resulted in widespread loss of Kac through histone deacetylase (HDAC) activity (Houston et al., 2020).

We sought to establish an effective model allowing for the study of BRD-mediated acetylation-dependent protein-protein interactions. Toward this goal, we created *de novo* Flp-In T-REx HEK293 cells lacking ACLY and characterized how rapid acetyl-CoA depletion could be combined with functional proteomics. Kac depletion in *ACLYΔ* cells was compatible with both fast proximity biotinylation (TurboID) and affinity purification (AP) coupled to mass spectrometry (MS). The *ACLYΔ* model allowed us to characterize both global and specific reorganizations of protein interaction networks in response to Kac changes.

## 2 Materials and Methods

### 2.1 Generation and Validation of ACLYΔ Cells

The genomic ablation of *ACLY* in Flp-In T-REx HEK293 cells was performed using CRISPR/Cas9 as previously reported (Ran et al., 2013). Briefly, two guide RNAs (gRNA) (see Supplementary Table S1A for sequences) were designed using CRISPOR via the UCSD Genome Browser (Concordet and Haeussler, 2018), and the oligos were annealed and ligated into BbsI-digested px459 V2.0. The resulting plasmids (2 μg) were transfected in Flp-In T-REx HEK293 cells using JetPrime (114-07; Polyplus-transfection, France, Illkirch) following the supplier’s instructions. The medium was replaced with fresh medium containing 2 μg/ml of puromycin 24 h after transfection to initiate the selection of cells expressing Cas9. For the next 2 days, the medium was refreshed as necessary to remove dead cells. The efficacies of the gRNAs for ACLY ablation were determined by analyzing the pool of puromycin-selected cells by western blot for ACLY and TIDE analysis (Brinkman et al., 2014). To isolate *ACLYΔ* clones, a limiting dilution of the pool of resistant cells was performed in a 96-well plate and individual clones were validated by sequencing and western blotting. CRISP-ID was used to genotype the clones based on Sanger sequencing traces (Dehairs et al., 2016).

### 2.2 Cell Line Production and Growth

The Flp-In T-REx HEK293 cell line and its subclones were cultured in Dulbecco’s modified Eagle’s medium (DMEM) supplemented with 10% fetal bovine serum (FBS; Wisent, Canada, Quebec, St-Bruno) and 1% penicillin/streptomycin (LS15140122; Gibco) and 0 or 20 mM sodium acetate (S2889; Sigma-Aldrich, Canada, Ontario, Oakville) in a humidified atmosphere with 5% CO_2_. Unless specified, Flp-In T-REx HEK293 *ACLYΔ* cell medium was always supplemented with 20 mM sodium acetate. For Kac depletion experiments, we used dialyzed FBS (dFBS; Wisent) sourced from the same lot as the complete FBS. For AP-MS, a construct encoding BRD9 (the bait; MGC clone # BC031484), was generated via Gateway cloning into pDEST 5′ 3×FLAG-pcDNA5-FRT-TO as in (Lambert et al., 2019), and BRD9 was stably expressed in T-REx Flp-In HEK293 cells as described in (Lambert et al., 2014). A stable line expressing 3×FLAG fused to green fluorescent protein (GFP) and parental Flp-In T-REx HEK293 cells were used as negative controls and processed in parallel to the baits. Stable cell lines were selectively grown in the presence of 200 μg/ml hygromycin until 80% confluent, then bait expression was induced via 1 μg/ml tetracycline for 24 h. The cells were harvested, pelleted at low speed, washed with ice-cold phosphate-buffered saline (PBS), and frozen at −80°C until purification. For TurboID experiments, Flp-In T-REx HEK293 cells were used and constructs encoding TurboID-tagged histone H2B and H3.1 were generated via Gateway cloning into pDEST 3′ TurboID-3×FLAG pcDNA5-FRT-TO as per (Lambert et al., 2015). TurboID-3×FLAG- and TurboID-GFP-expressing cells were used as negative controls and processed in parallel. Cells were maintained in biotin-depleted medium (generated by incubating 50 ml FBS aliquots with 100 μl of sterile, PBS-washed streptavidin slurry overnight at 4°C prior to preparing the medium) for three passages prior to TurboID. TurboID stable cell lines were selectively grown in the presence of 200 μg/ml hygromycin until 80% confluent, when expression was induced via 1 μg/ml tetracycline for 23 h. Then, the cells were treated with 50 μM biotin for 1 h and harvested.

### 2.3 Chemical Inhibitors

Details regarding all chemical inhibitors used in this study can be found in Supplementary Table S1B.

### 2.4 Clonogenic Assays

Cells were seeded at 5,000 or 10,000 cells per well in 24- or 12-well poly-L-lysine (Sigma-Aldrich; P4707)-coated plates, respectively, in DMEM supplemented with 1, 5, or 10% FBS (Wisent), 1% antibiotics (penicillin/streptomycin; Gibco), and 0, 2, or 20 mM sodium acetate (Sigma-Aldrich; S2889). Plates were incubated in a humidified atmosphere with 5% CO_2_ for 6 days, then stained with crystal violet. BRD inhibitors used in some experiments were dissolved in dimethyl sulfoxide, the volume of which constituted a maximum 0.1% (v/v) of the medium.

### 2.5 Immunofluorescence

Cells were seeded into a 12-well plate containing poly-L-lysine-coated coverslips in complete medium and grown for 48 h in the presence of 20 mM sodium acetate for 48 h, induced with 1 μg/ml tetracycline for 24 h, then incubated with dFBS or complete FBS for 1 h. Cells were fixed with 4% paraformaldehyde in PBS for 15 min at room temperature and then stained for Kac, GFP, or phalloidin as detailed in Supplementary Table S1B. Image stacks were acquired on a Leica DMI 6000 B inverted microscope with a Yokogawa CSU10 confocal unit at 63×. Z stacks were collected, deconvolved using Volocity (Quorum Technologies), and shown as intensity projections. Images were cropped using Adobe Photoshop. For all quantitatively compared images, identical imaging conditions (including exposure times) were used.

### 2.6 Immunoblotting

For western blot analysis, 10–50 μg of protein was resolved by sodium dodecyl sulfate-polyacrylamide gel electrophoresis, transferred to nitrocellulose, and blocked in Tris-buffered saline containing either 5 mg/ml non-fat milk or bovine serum albumin and 1% Tween-20 for 1 h at room temperature. Antibodies and their conditions can be found in Supplementary Table S1B. Detection on film was performed by chemiluminescence using the Clarity Western ECL Substrate (Bio-Rad; #1705061). Films were scanned and figures were assembled using Adobe Photoshop and Adobe Illustrator.

### 2.7 Affinity Purification

The GFP AP-MS protocol was adapted from (Lambert et al., 2014), with slight modifications. Stable cells from two 150 mm plates were pelleted, frozen, and lysed in 1.5 ml ice-cold low salt lysis buffer (50 mM HEPES-NaOH pH 8.0, 100 mM KCl, 2 mM ethylenediaminetetraacetic acid, 0.1% NP-40, and 10% glycerol, with 1 mM phenylmethylsulfonyl fluoride, 1 mM dithiothreitol, and Sigma-Aldrich protease inhibitor cocktail (1:500) added immediately prior to processing). To aid with lysis, the cells were frozen on dry ice, thawed in a 37°C water bath, and returned to ice. The samples were sonicated in 30 s bursts with 2 s pauses at 35% amplitude using a Q125 sonicator (QSONICA). Then, turbonuclease (100 units, Sigma-Aldrich, T4332) was added and the lysates were rotated at 4°C for 1 h. The lysates were centrifuged at 20,817 × g for 20 min at 4°C and the supernatant was added to tubes containing 25 μl of a 50% slurry of GFP-Trap magnetic agarose beads (ChromoTek, Germany; gtma-10) prewashed with lysis buffer. GFP immunoprecipitation was performed at 4°C for 2 h with rotation. Beads were pelleted by centrifugation (1,000 rpm for 1 min) and magnetized, and the unbound lysate was aspirated and kept for analysis. The beads were demagnetized, washed with 1 ml lysis buffer, and remagnetized to aspirate the buffer. The beads were then washed with 1 ml of 20 mM Tris-HCl (pH 8.0) containing 2 mM CaCl_2_ and any excess buffer was removed by centrifuging the beads, magnetizing, and removing the liquid with a pipette. The dry magnetic beads were demagnetized, resuspended in 7.5 μl of 20 mM Tris-HCl (pH 8.0) containing 750 ng of trypsin (Sigma-Aldrich, T6567), and incubated overnight at 37°C with agitation. After this initial incubation, the beads were magnetized and the supernatant was transferred to a fresh tube. Another 250 ng of trypsin was added to the supernatant for further digestion without agitation for 3–4 h. Samples were acidified with formic acid to a final concentration of 2% and desalted using homemade C_18_ Stage Tips as previously described (Rappsilber et al., 2007). Peptide samples were stored at -80°C until MS analysis.

### 2.8 TurboID

The TurboID protocol was adapted from (Lambert et al., 2015), with slight modifications. Cells from two 150 mm plates were pelleted, frozen, and thawed in 1.5 ml ice cold radioimmunoprecipitation buffer [50 mM Tris-HCl (pH 7.5), 150 mM NaCl, 1% NP-40, 1 mM ethylenediaminetetraacetic acid, 1 mM ethylene glycol tetraacetic acid, 0.1% sodium dodecyl sulfate, and 0.5% sodium deoxycholate]. Phenylmethylsulfonyl fluoride (1 mM), dithiothreitol (1 mM), and Sigma-Aldrich protease inhibitor cocktail (1:500) were added immediately before use. The lysates were sonicated, treated with turbonuclease, and centrifuged as described in Section 2.7. For each sample, 60 μl of Streptavidin Sepharose High Performance Affinity Chromatography Medium (Cytiva, Cat 17-5113-01) was prewashed three times with 1 ml of lysis buffer, by pelleting the beads with gentle centrifugation and aspirating the supernatant before adding the next wash. Biotinylated proteins were captured on pre-washed streptavidin beads for 3 h at 4°C with rotation. The beads were gently pelleted and then washed twice with 1 ml of radioimmunoprecipitation buffer and three times with 1 ml of 50 mM ammonium bicarbonate (pH 8.0). Following the final wash, the beads were pelleted and any excess liquid was aspirated. Beads were resuspended in 100 μl of 50 mM ammonium bicarbonate, and 1 μg of trypsin solution was added. The samples were incubated overnight at 37°C with lateral shaking and then an additional 1 μg of trypsin was added, followed by an additional 2–4 h of incubation. The beads were pelleted and the supernatant was transferred to a fresh tube. The beads were rinsed twice with 100 μl of high-performance liquid chromatography-grade acetonitrile and the wash fractions were combined with the supernatant. The peptide solution was acidified with 50% formic acid to a final concentration of 2% and the samples were dried in a SpeedVac. Tryptic peptides were stored at −80°C until MS analysis.

### 2.9 Experimental Design and Statistical Rationale for MS Experiments

For each analysis, at least two biological replicates of each bait were processed independently, with negative controls included in each batch of processed samples. The order of sample acquisition on the LC-MS/MS system was randomized. Statistical scoring was performed against the negative controls using Significance Analysis of INTeractome [(Teo et al., 2014); SAINTexpress 3.6.1] as defined in Supplementary Tables S2A,B. The average SAINTexpress score was used to determine the Bayesian false discovery rate (FDR), which requires a high confidence interaction to be detected in both biological replicates to pass our 1% FDR significance threshold.

### 2.10 Data-Dependent Acquisition MS

MS analyses were performed at the Proteomics Platform of the Quebec Genomics Center. Peptide samples were separated by online reversed-phase nanoscale capillary liquid chromatography and analyzed by electrospray MS/MS. The experiments were performed with a Dionex UltiMate 3000 RSLCnano chromatography system (Thermo Fisher Scientific) connected to an Orbitrap Fusion mass spectrometer (Thermo Fisher Scientific) equipped with a nanoelectrospray ion source. Peptides were trapped at 20 μl/min in loading solvent (2% acetonitrile, 0.05% TFA) on an Acclaim 5 μm PepMap 300 μ-Precolumns Cartridge Column (Thermo Fisher Scientific) for 5 min. Then, the precolumn was switched online with a laboratory-made 50 cm × 75 μm internal diameter separation column packed with ReproSil-Pur C_18_-AQ 3-μm resin (Dr. Maisch HPLC) and the peptides were eluted with a linear gradient of 5–40% solvent B (A: 0,1% formic acid, B: 80% acetonitrile, 0.1% formic acid) over 90 min at 300 nl/min. Mass spectra were acquired in data-dependent acquisition mode using Thermo XCalibur software version 3.0.63. Full scan mass spectra (350–1,800 m*/z*) were acquired in the Orbitrap using an AGC target of 4e5, a maximum injection time of 50 ms, and a resolution of 120,000. Internal calibration using lock mass on the *m/z* 445.12003 siloxane ion was used. Each MS scan was followed by acquisition of the fragmentation spectra of the most intense ions for a total cycle time of 3 s (top speed mode). The selected ions were isolated using the quadrupole analyzer in a window of 1.6 m*/z* and fragmented by higher energy collision-induced dissociation at 35% collision energy. The resulting fragments were detected by the linear ion trap in rapid scan rate with an AGC target of 1e4 and a maximum injection time of 50 ms. Dynamic exclusion of previously fragmented peptides was set for a period of 20 s and a tolerance of 10 ppm.

### 2.11 Protein Identification

MS data were stored, searched, and analyzed using the ProHits laboratory information management system (Liu et al., 2016). Thermo Fisher scientific RAW mass spectrometry files were converted to mzML and mzXML using ProteoWizard [3.0.4468; (Kessner et al., 2008)]. The mzML and mzXML files were then searched using Mascot (v2.3.02) and Comet (v2012.02 rev.0) against the RefSeq database (version 57, 30 January 2013) acquired from NCBI, containing 72,482 human and adenovirus sequences supplemented with “common contaminants” from the Max Planck Institute (http://lotus1.gwdg.de/mpg/mmbc/maxquant_input.nsf/7994124a4298328fc125748d0048fee2/$FILE/contaminants.fasta), and the Global Proteome Machine (GPM; http://www.thegpm.org/crap/index.html). Charges of +2, +3, and +4 were allowed and the parent mass tolerance was set at 12 ppm while the fragment bin tolerance was set at 0.6 amu. Deamidated asparagine and glutamine and oxidized methionine were allowed as variable modifications. The results from each search engine were analyzed through the Trans-Proteomic Pipeline (v4.6 OCCUPY rev 3) (Deutsch et al., 2015) via the iProphet pipeline (Shteynberg et al., 2011). To identify significant interaction partners, we used SAINTexpress [(Teo et al., 2014); version 3.6.1] using default parameters. The results of these analyses can be found in Supplementary Tables S2A,B.

### 2.12 MS Data Visualization and Archiving

We used ProHits-viz (Knight et al., 2017) to generate scatter and dot plots. To enhance the illustrations, individual nodes or dots were manually arranged in some figures. All MS files used in this study were deposited to MassIVE (http://massive.ucsd.edu) and can be accessed at ftp://MSV000088171@massive.ucsd.edu and ftp://MSV000088402@massive.ucsd.edu. Additional details (including MassIVE accession numbers and FTP download links) can be found in Supplementary Table S2C.

### 2.13 Extracellular Flux Analyses

Oxygen consumption rates (OCRs) and extracellular acidification rates (ECARs) were measured using a Seahorse XFe96 Analyzer (Agilent). Briefly, 15,000 cells/well were seeded in a 96-well Seahorse microplate in Seahorse XF RPMI-1640 medium supplemented with 20 mM sodium acetate. After an 18 h incubation, the medium was replaced with Seahorse XF RPMI Medium supplemented with 10 mM glucose, 2 mM glutamine, 1 mM sodium pyruvate, and penicillin (100 U/ml)/streptomycin (100 μg/ml) prior to a 1 h equilibration period in a CO_2_-free incubator at 37°C. After equilibration, the microplate was then loaded into the instrument for OCR and ECAR analyses. Three injections were performed sequentially for the mitochondrial stress test, with each drug (3.8 μM oligomycin, 0.6 μM carbonylcyanide-p-trifluoromethoxyphenylhydrazone (FCCP), 1.2 μM antimycin A, and 3.6 μM rotenone) injected following three measurements of the OCR and ECAR. At the end of the assay, for normalization, the cells were quantified by CyQUANT Proliferation Assay as previously described (Lacouture et al., 2021). Student’s *t*-test was used to evaluate statistical significance, with *p* < 0.05 considered significant.

### 2.14 Fluorometric Acetyl-CoA Assays

Acetyl-CoA was quantified using the Acetyl-Coenzyme A Assay Kit (MAK039; Sigma-Aldrich) according to the manufacturer’s instructions with slight modifications. Briefly, parental and *ACLYΔ* HEK293 cells were seeded in 10 cm plates in DMEM supplemented with 10% FBS (Wisent), antibiotics (penicillin/streptomycin; Gibco), and 20 mM sodium acetate (Sigma-Aldrich; S2889) in a humidified atmosphere with 5% CO_2_ for 48 h to a confluence of ∼80%. For Kac depletion, dFBS (Wisent) sourced from the same lot as the complete FBS was added for 1 h. Cells were washed with warm 1× PBS and harvested by mechanical dissociation following the indicated treatments. Samples were deproteinized by adding 2 μl of 1 M perchloric acid per mg of protein and incubating on ice for 30 min with intermittent vortexing. Samples were then centrifuged at 10,000 × g for 10 min and the supernatants were transferred to fresh tubes and neutralized (pH 6–8) with 3 M potassium bicarbonate (Sigma-Aldrich; 60339). Samples corresponding to ∼0.23 mg of protein sample prior to deproteinization (50 μl) were used in each analysis. The fluorescence intensity of each sample was measured at excitation and emission wavelengths of 535 and 587 nm, respectively, on a Synergy H1 Hybrid Multi-Mode Reader (BioTek Instruments). Ordinary two-way analysis of variance was used to evaluate statistical significance, with *p* < 0.05 considered significant.

### 2.15 Targeted Acyl-CoA Quantification by LC-Multiple Reaction Monitoring/MS

Nine serially diluted reference standards (0.0001–20 μM) were prepared for each of the targeted CoA metabolites (see Supplementary Table S2D for the complete list) in an internal standard solution containing ^13^C_3_-malonyl CoA in 1:4 water-methanol (v/v). Flp-In T-REx HEK293 parental or *ACLYΔ* g1_1 cells were grown to ∼75% confluence in complete DMEM supplemented with 20 mM sodium acetate. The cells were washed with warm PBS and treated with DMEM containing 10% dFBS with or without 20 mM sodium acetate for 1 h. The cells were then trypsinized and counted. Three aliquots of 10 million cells were pelleted, the supernatants were removed, and the pellets were frozen on dry ice and stored at −80°C. At the time of analysis, cells were thawed on ice and 80 μl of pH ∼7 Tris buffer was added to each sample. The samples were then lysed with two 3 mm metal balls on a MM 400 mill mixer (Retsch) at a shaking frequency of 30 Hz, then 500 μl of a 3:1 methanol-chloroform solution was added. The mixture was vortexed for 1 min, sonicated for 30 s, then placed at −20°C for 1 h before centrifugation at 21,000 × *g* and 5°C for 10 min. A 100 μl aliquot of the supernatant was mixed with 50 μl of the internal standard solution and then dried under a gentle nitrogen gas flow. The dried residue was reconstituted in 50 μl of 80% methanol and centrifuged for clarification. Aliquots of each sample and each standard (10 μl) were injected into a Waters UPLC system coupled to a SCIEX QTRAP 6500 + MS instrument in negative-ion mode. LC separation was performed on a C_18_ column (2.1 × 100 mm, 1.8 μm) using ammonium bicarbonate buffer (A) and mixed acetonitrile-isopropanol (B) as the mobile phase for binary-solvent elution with an efficient gradient of 1–90% B over 18 min at 50°C and 0.3 ml/min. The concentrations of detected compounds were calculated by interpolating the constructed linear-regression curves of individual compounds with the analyte-to-internal standard peak area ratios measured from each sample solution. The complete results of this analysis are found in Supplementary Table S2D.

## 3 Results

### 3.1 Establishment of the *ACLYΔ* Model

**FIGURE 2:**
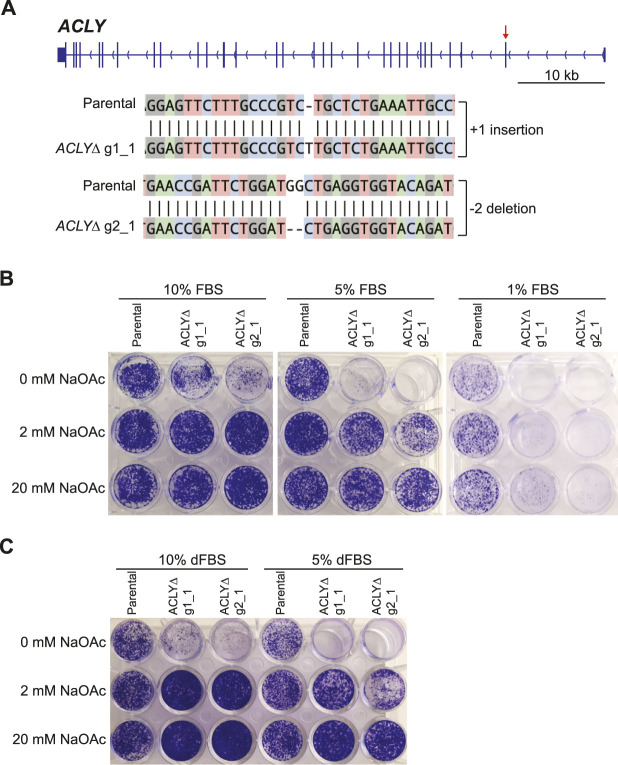
Establishment of a nutritional model enabling Kac signaling modulation. **(A)** Overview of *ACLY* and sequencing data from the HEK293 *ACLYΔ* clones selected for further characterization. The red arrow indicates the first exon, which was targeted using two independent gRNAs. Alignments were generated using CRISP-ID (Dehairs et al., 2016). **(B)** Clonogenic assays of HEK293 *ACLYΔ* and parental cells incubated with and without NaOAc in medium containing 1, 5, or 10% FBS. **(C)** Clonogenic assay of HEK293 *ACLYΔ* and parental cells incubated with and without NaOAc in medium containing 5 or 10% dFBS.

Kac sites are key effectors of chromatin structure and function, and act by neutralizing the overall positive charge of histone proteins and recruiting specific regulators—notably, those containing BRDs. To investigate Kac-dependent protein-protein interactions, we sought to establish an *in vitro* model enabling the effective modulation of Kac marks. Recently, Liu et al. and Houston et al. reported that *ACLY* KO renders cells dependent on exogenous acetate by preventing the conversion of mitochondrial citrate to acetyl-CoA and oxaloacetate in the cytoplasm and nucleus (Figure 1) (Liu et al., 2018; Houston et al., 2020). Targeting the first exon of *ACLY* with two distinct gRNAs, we successfully established *ACLY* KO Flp-In T-REx HEK293 cell lines (HEK293 *ACLYΔ*), but only when the medium was supplemented with sodium acetate (NaOAc) during selection (Figure 2A). Using clonogenic assays, we found that in medium containing 10% FBS, HEK293 *ACLYΔ* cells were viable over 6 days but exhibited a drastically reduced growth rate, and medium supplementation with 2 or 20 mM NaOAc rescued this phenotype (Figure 2B). Reducing the FBS in the culture medium to 1 or 5% resulted in the death of most HEK293 *ACLYΔ* cells (Figure 2B), consistent with previous reports that FBS contains low amounts of acetate that may contribute to the survival of the KO cells (Kamphorst et al., 2014; Zhao et al., 2016; Houston et al., 2020). Dialyzing FBS to remove acetate and other small molecules prevented the survival of most HEK293 *ACLYΔ* cells in the absence of exogenous NaOAc (Figure 2C). The use of ACLY chemical inhibitors SB-204990, NDI-091143, and ETC-1002 did not mimic *ACLY* KO in our clonogenic assay, reinforcing the need for our genetic approach to specifically and completely inhibit ACLY activity (Supplementary Figure S1).

### 3.2 Characterization of the *ACLYΔ* Model

**FIGURE 3:**
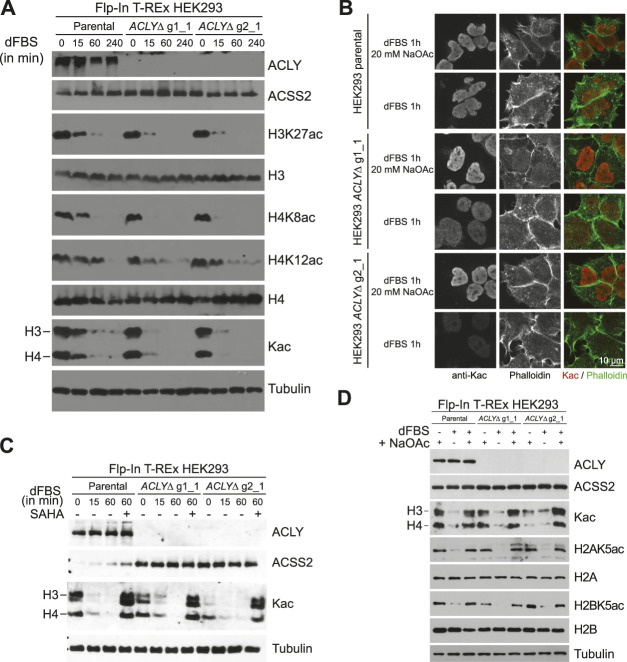
Acetate depletion in HEK293 *ACLYΔ* cells decreases Kac levels rapidly. **(A)** Western blot analysis of ACLY, ACSS2, and histone Kac levels during a dFBS time-course of HEK293 *ACLYΔ* and parental cells. **(B)** Immunofluorescence of HEK293 *ACLYΔ* and parental cells incubated with and without NaOAc in medium containing 10% dFBS for 1 h. Cells were stained with anti-Kac antibodies and phalloidin. **(C)** Western blot analysis of ACLY, ACSS2, and histone Kac levels during a dFBS time-course of HEK293 *ACLYΔ*, and parental cells with or without 5 μM suberanilohydroxamic acid (SAHA) treatment. **(D)** Western blot analysis of ACLY, ACSS2, and histone Kac levels following dFBS treatment of HEK293 *ACLYΔ* and parental cells with or without NaOAc supplementation.

Having established an effective, fast, scalable, and affordable model in which to investigate fundamental mechanisms affected by Kac level modulation, we first quantified Kac marks following acetyl-CoA depletion by western blot. Parental and *ACLYΔ* cells were grown in complete medium supplemented with 20 mM NaOAc. Subsequent transfer to DMEM with 10% dFBS and no NaOAc resulted in drastic reductions in histone acetylation in as little as 15 min (Figure 3A). HEK293 *ACLYΔ* cells lost their Kac marks more rapidly than their parental counterparts. Importantly, parental cells maintained a low level of histone acetylation that was not apparent for most Kac marks tested in the HEK293 *ACLYΔ* cells (Figure 3A). Immunofluorescence analysis of *ACLYΔ* cells treated with 10% dFBS with and without 20 mM NaOAc for 1 h revealed clear reductions in the levels of acetylated nuclear proteins that were not observed in parental cells (Figure 3B). HDAC inhibition with 5 μM suberanilohydroxamic acid prevented Kac loss after 10% dFBS treatment (Figure 3C). This is consistent with the catabolism of Kac marks by HDACs to regenerate acetyl-CoA previously reported in cells lacking ACLY (Houston et al., 2020). Next, we sought to confirm that acetate supplementation could directly overcome the catabolism of Kac marks induced by dFBS treatment. We treated HEK293 parental and *ACLYΔ* cells with medium containing 10% dFBS with or without 20 mM NaOAc for 1 h, and found that NaOAc supplementation prevented the loss of Kac marks (Figure 3D).

**FIGURE 4:**
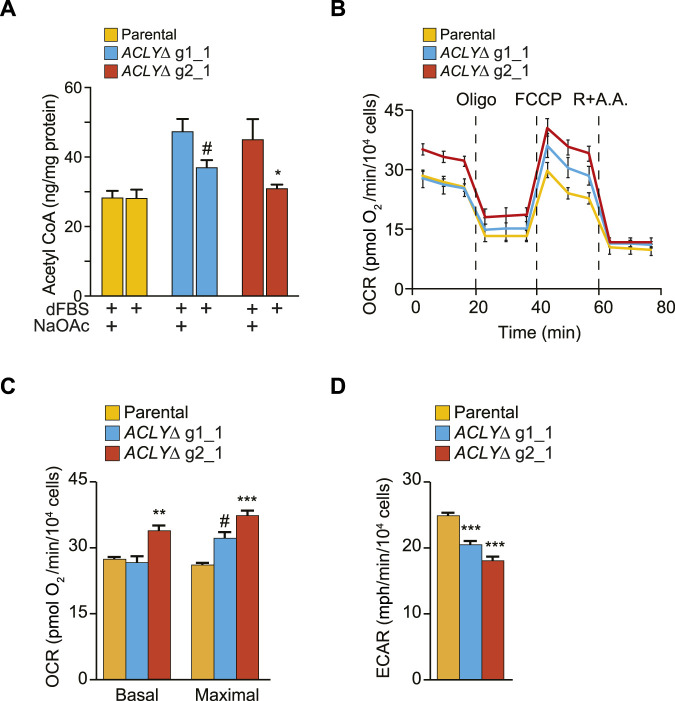
HEK293 *ACLYΔ* cells have altered metabolism. **(A)** Quantification of acetyl-CoA in HEK293 *ACLYΔ* and parental cells incubated with and without NaOAc in medium containing 10% dFBS for 1 h using a fluorometric assay. **(B)** OCRs were measured and normalized to cell numbers. Following basal respiration measurements, drugs that selectively target specific components of the electron transport chain were sequentially injected. Oligomycin inhibits ATP synthase, blocking oxygen consumption coupled to ATP production. FCCP disrupts the proton gradient, thereby inducing maximal respiration. Finally, antimycin A (a complex III inhibitor) and rotenone (a complex I inhibitor) were simultaneously injected to completely block mitochondrial respiration, leaving only non-mitochondrial respiration. **(C)** Measurements of basal and maximal respiration in parental and *ACLYΔ* cells. **(D)** Basal ECARs in parental and *ACLYΔ* cells. For **(B–D)**, results from one representative experiment are shown, with 12 replicates/condition, out of at least three independent experiments. ***p < 0.001; **p < 0.01; *p < 0.05; ^#^ *p* < 0.12.

To determine if the observed reduction in Kac levels (Figure 3A) was concomitant with a general metabolic rewiring, we first used a fluorometric assay to quantify the acetyl-CoA levels in parental and *ACLYΔ* HEK293 cells following a 1 h dFBS treatment (Figure 4A). We found that acetyl-CoA levels were significantly reduced in *ACLYΔ* g2_1 cells in the absence of NaOAc supplementation. A similar trend was observed for the *ACLYΔ* g1_1 cells; however, it did not reach significance. Next, we quantified common acyl-CoA metabolites in parental and *ACLYΔ* cells treated with 10% dFBS with or without 20 mM NaOAc for 1 h, using a targeted LC-MS approach to quantify free CoA and C_2_ to C_26_ acyl-CoA metabolites (Supplementary Table S2D). We observed similar trends regarding the reduced levels of acetyl-CoA in *ACLYΔ* g1_1 compared to the parental cells in these analyses. We did find a significant reduction of free CoA in *ACLYΔ* cells when their medium was supplemented with 20 mM NaOAc, supporting the enhanced production of acyl-CoA metabolites by the ACSS2 pathway in these cells (Supplementary Table S2D).

ACLY generates acetyl-CoA using citrate, the first intermediate of the tricarboxylic acid cycle that is coupled with mitochondrial respiration. Therefore, we directly investigated whether our *ACLYΔ* clones used compensatory mechanisms to increase tricarboxylic acid cycle activity and promote citrate synthesis by measuring their OCRs with an extracellular flux analyzer. Basal respiration was unchanged between parental and *ACLYΔ* g1_1 cells, but was significantly increased in *ACLYΔ* g2_1 cells (Figures 4B,C). Following the mitochondrial stress test, addition of FCCP also revealed a significantly higher maximal respiration in *ACLYΔ* g2_1 cells, with a similar trend observed in *ACLYΔ* g1_1 cells (Figure 4B). In parallel to OCR, the XFe96 instrument also measures the ECAR, a proxy for lactate secretion (aerobic glycolysis). We observed that KO of *ACLY* led to ∼20% (*p* < 0.001) and 25% (*p* < 0.001) decreases in ECAR in the *ACLYΔ* g1_1 and g2_1 lines, respectively (Figure 4D). These results indicate that *ACLY* KO alters the cell’s bioenergetic capacity. Together, these results confirm our capacity to decrease acetylated histone levels via a 1 h dFBS treatment in a HDAC-dependent manner without drastically altering the global metabolism of HEK293 *ACLYΔ* cells, despite modulating their bioenergetic capacity.

### 3.3 Global Chromatin Rearrangements Following Kac Depletion

**FIGURE 5:**
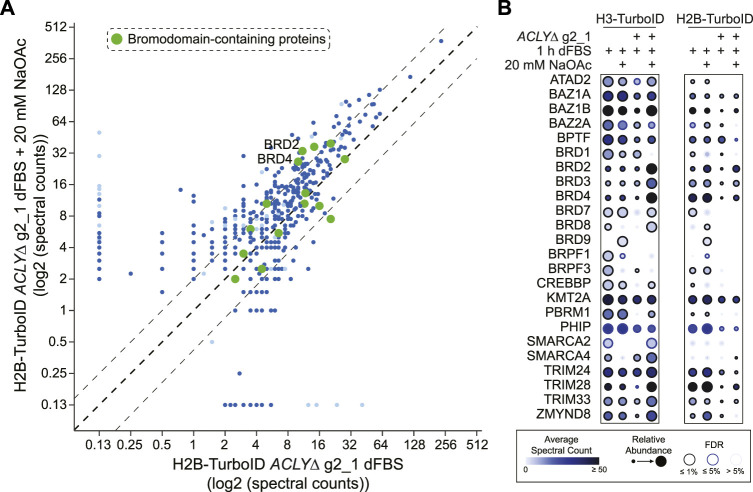
Global chromatin rearrangements following Kac depletion. **(A)** Bait versus bait scatter plot of H2B-TurboID in HEK293 *ACLYΔ* g2_1 cells comparing dFBS treatments with and without 20 mM NaOAc. BRD-containing proteins are colored in green with significant proximity partners (FDR ≤1%) shown in dark blue and those with an FDR between 1 and 5% in light blue. The black dashed diagonal line indicates equal abundance for both conditions, with relative two-fold enrichment indicated by gray dashed lines on either side. **(B)** Dot plot of BRD-containing proteins identified as significant proximity partners (FDR ≤1%) of TurboID-tagged H3 and H2B in HEK293 *ACLYΔ* g2_1 cells treated with dFBS for 1 h with and without 20 mM NaOAc. See insert legend for details.

Having established effective conditions for Kac depletion in our *ACLYΔ* model, we sought to investigate the consequences associated with Kac depletion at the level of chromatin. To do so, we performed fast proximity biotinylation assays of the histones H2B and H3.1, tagged with the abortive biotin ligase TurboID. We reasoned that these core histone interactomes would highlight reorganizations of the chromatin composition in a time-frame compatible with our model. We quantified the biotinylated proteins generated by TurboID-tagged histone H2B and H3 in cells treated with dFBS with or without 20 mM NaOAc, and identified hundreds of proteins whose associations with histones were influenced by Kac levels. Next, we performed a SAINTexpress analysis of the data using GFP-TurboID as a control (Supplementary Table S2A). We observed that the reduction in Kac levels generated by removing NaOAc supplementation was correlated with reductions in the proteins proximal to H2B and H3 in our TurboID assay (Figure 5A). Focusing on the 24 BRD-containing proteins identified at an FDR of ≤1% in at least one sample, we observed that the levels of co-purified BRD-containing proteins were stable in parental Flp-In T-REx HEK293 cells (Figure 5B). Conversely, in *ACLYΔ* g2_1 cells, depletion of Kac significantly reduced the levels of multiple BRD-containing proteins, notably the bromodomain and extra-terminal motif (BET) protein family members BRD2, BRD3, and BRD4 (Figure 5B). Supplementation with 20 mM NaOAc was sufficient to overcome this reduction. Intriguingly, some BRD-containing proteins were insensitive to Kac depletion (e.g., BAZ2A), suggesting that other domains mediate their interactions with the chromatin. This is reminiscent of our previous fluorescence recovery after photobleaching assays, in which disrupting BRD-dependent interactions was not necessarily sufficient to enhance the mobility of some BRD-containing proteins (Philpott et al., 2014). Together, these analyses demonstrate that Kac depletion via a short dFBS treatment enables the molecular investigation of BRD-containing proteins that are primarily anchored to the chromatin through their BRD.

### 3.4 Use of the *ACLYΔ* Model in Interactome Mapping Experiments

**FIGURE 6:**
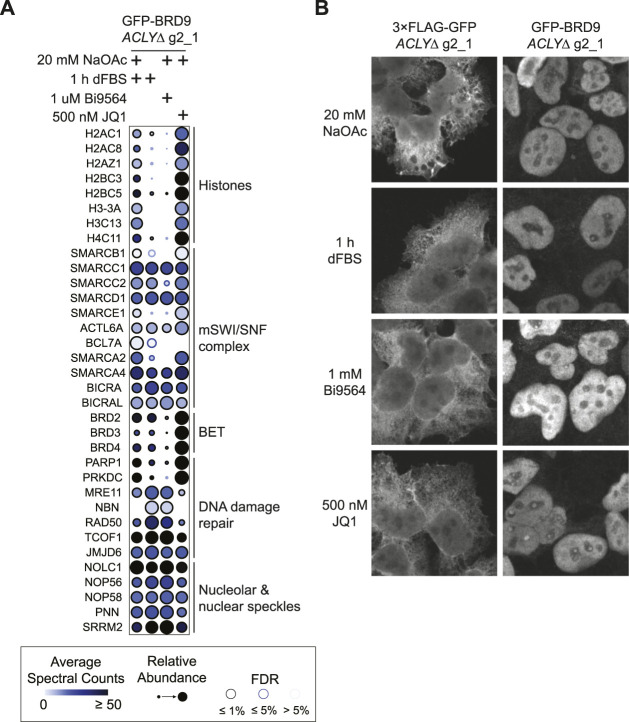
Reorganization of the BRD9 interactome upon Kac depletion. **(A)** Dot plot of selected interaction partners of GFP-BRD9 identified by AP-MS of HEK293 *ACLYΔ* g2_1 cells treated with or without 20 mM NaOAc and treated with 10% dFBS, 1 μM Bi9564, or 500 nM JQ1 for 1 h. **(B)** Immunofluorescence of GFP-BRD9 and GFP-3 ×FLAG in HEK293 *ACLYΔ* g2_1 cells incubated with and without NaOAc in medium containing 10% dFBS for 1 h. Cells were immunostained for GFP.

To directly demonstrate the utility of our nutrient-based model for the characterization of Kac readers, we next investigated BRD9, a BRD-containing protein that was weakly detected in our TurboID assay (Figure 5). BRD9 is a core subunit of the non-canonical BAF complex (Mashtalir et al., 2018). We performed affinity purification of GFP-tagged BRD9 from *ACLYΔ* cells treated with dFBS with or without 20 mM NaOAc in parallel to cells treated with the BRD9 BRD inhibitor Bi9564 (Martin et al., 2016) or the pan-BET BRD inhibitor JQ1 (Filippakopoulos et al., 2010). SAINTexpress analysis revealed that BRD9 established an extensive protein interaction network at the level of the chromatin (Figure 6A; Supplementary Table S2B). Removal of NaOAc reduced the levels of histones co-purifying with BRD9 similarly to Bi9564; this was not observed with the BET family BRD inhibitor JQ1, which does not target BRD9 directly (Figure 6A). Interestingly, we observed that dFBS and Bi9564 treatment resulted in enhanced interactions between BRD9 and numerous nucleolar and nuclear speckle proteins, notably the MRN complex (composed of MRE11, NBN and RAD50); treacle ribosome biogenesis factor 1 (TCOF1); and jumonji domain containing 6, arginine demethylase and lysine hydroxylase (JMJD6); which are known to promote DNA damage repair in the nucleus (Larsen et al., 2014; Fages et al., 2020; Mooser et al., 2020). Immunofluorescence analysis of GFP-BRD9 and a GFP-3 ×FLAG control in *ACLYΔ* cells treated with dFBS with or without 20 mM NaOAc showed that BRD9 constitutively localizes in the nucleolus, supporting our AP-MS results (Figures 6B,C). Together, our results support a model in which Kac levels regulate the BRD9 interactome beyond histone proteins, and reinforces the value of our *ACLYΔ* model in characterizing BRD-dependent interactions.

## 4 Discussion

Here, we report the establishment of an *in vitro* model enabling the rapid modulation of Kac signaling and its use to investigate BRD-dependent protein-protein interactions. Using Flp-In T-REx HEK293 cells, a common model for functional proteomics studies (Go et al., 2021), we have shown that a brief (1 h) removal of acetate from the culture medium was sufficient to drastically reduce the levels of acetylated proteins (Figure 3). As first reported by Houston et al. (Houston et al., 2020), this occurs through the catabolism of Kac by HDACs to regenerate the nucleocytoplasmic pool of acetyl-CoA (Figure 3C). Critically, metabolic alterations were limited within this time frame (Figure 4; Supplementary Table S2D).

We investigated whether chemical ACLY inhibition was sufficient to make cells dependent on the ACSS2 pathway for acetyl-CoA production. Using three distinct ACLY inhibitors (Supplementary Figure S1B), we were unable to recapitulate the phenotypes observed by KO, raising an interesting question about the mode of action of these compounds. This was not unforeseen for ETC-1002 (bempedoic acid) since the free acid activates AMP-activated protein kinase (AMPK) while its coupling to CoA by very long-chain acyl-CoA synthetase (SLC27A2) enables effective ACLY inhibition (Pinkosky et al., 2013). Still, the failure of acetate supplementation to overcome co-treatment with SB-204990 or the recently described NDI-091143 suggest that these molecules have cellular impacts beyond modulating acetyl-CoA levels that are not addressed by our supplementation strategy. An in-depth characterization of the metabolome of these cells may help answer these unresolved questions.

In the context of acute myeloid leukemia tumors, Jiang et al. showed that AMP-activated protein kinase KO reduces tumor initiation and Kac levels in *in vivo* models in an acetate-dependent fashion (Jiang et al., 2019). Furthermore, they showed that reduced Kac levels minimize the retention of BET family BRD-containing proteins to key acetylated binding sites (Jiang et al., 2019), notably at disease-specific *MYC* enhancers (Delmore et al., 2011). In fact, the reduced Kac levels in these models due to AMPK KO sensitize cells to BRD inhibitors targeting BET proteins (Jiang et al., 2019; Traer, 2019). This is consistent with our proximity biotinylation assays of histones H2B and H3, which showed that the associations of BET proteins with chromatin were sensitive to Kac levels (Figure 5). Similarly, butyrylation or crotonylation of H4K5 (as opposed to its acetylation) via the aberrant activation of FAST kinase domains 1 in acute lymphoblastic leukemia models reduce BRD4’s association with the chromatin (Gao et al., 2021). In our targeted characterization of BRD9, we observed a switch in its interaction partners consistent with these observations (Figure 6B). With high Kac levels, BRD9 favored interactions with histones and BET proteins, as we recently reported (Lambert et al., 2019). Following Kac depletion by dFBS treatment these interactions were reduced, while BRD9’s interactions with mSWI/SNF subunits remained mostly unchanged (Figure 6B). BRD9 binding to poly-acetylated histone H4 was recently reported to stimulate the chromatin remodeling activity of the non-canonical BAF complex, while inhibiting its BRD abrogated this process (Mashtalir et al., 2021). Synergy between Kac levels, mSWI/SNF activity, and BRD9 inhibition thus appears to be present in many cellular contexts. We are currently investigating the molecular mechanisms underlying these processes in greater detail.

Recently, independently inherited variants in *ACLY* were investigated to determine their impacts on lipid and lipoprotein levels as well as cancer and cardiovascular events in a large cohort of ∼650,000 participants (Ference et al., 2019). Nine variants were associated with reduced circulating lipoproteins, cholesterols, triglycerides, and phospholipids (Ference et al., 2019). These changes were reflected by a reduced risk of major cardiovascular events for participants with these *ACLY* variants, mimicking its inhibition (Ference et al., 2019). A decreased risk of lung cancer was also reported, although with weak statistical evidence (Ference et al., 2019). A concurrent clinical trial investigated the impact of the ACLY inhibitor ETC-1002 (bempedoic acid) on patients with atherosclerotic cardiovascular disease, heterozygous familial hypercholesterolemia, or both (Ference et al., 2019). In a trial involving over 2,200 patients, ETC-1002 treatment did not lead to higher incidence of overall adverse events when combined with statin therapy (Ray et al., 2019). Critically, the combination therapy resulted in significantly lower low-density lipoprotein cholesterol (Ference et al., 2019). Based on these findings, ETC-1002 was approved for the treatment of hypercholesterolemia in 2020 (Markham, 2020). While ETC-1002 monotherapies have had no major impacts on cancer incidence, its overall well-tolerated profile suggests its promise in combination therapies with numerous anti-cancer drugs, including BRD inhibitors.

A potential limitation of the *ACLYΔ* model is the activation of the p53 pathway and disruption of ribosomal RNA production upon prolonged Kac depletion (Houston et al., 2020). To circumvent these issues, we used a short acetate depletion period. Fortunately, we did not observe signs of p53 activation in our results. Prolonged culture in the presence of acetate could also lead to their adaptation via ACSS2 upregulation, since *ACLYΔ* cells rely on it to produce the nucleocytoplasmic pool of acetyl-CoA. This is consistent with our observation that in some of our assays, slightly different responses were observed for our two *ACLYΔ* clones. In addition, we observed that *ACLYΔ* cells had a higher basal level of acetyl-CoA level compared to the parental cells which may be due to enhanced ACSS2 expression. Therefore, care should be taken to match the passage numbers of the parental and *ACLYΔ* cells across all experiments, in addition to testing multiple clones to minimize these potential issues.

In conclusion, we have established an inexpensive and flexible cellular model allowing the study of Kac-dependent protein interaction networks. Using it, we have shown that the loss of Kac marks remodels the composition of the chromatin and the protein-protein interactions of BRDs. We expect our model to further the functional characterization of BRD-containing proteins across distinct cellular contexts.

## Data Availability

The datasets presented in this study can be found in online repositories. The names of the repository/repositories and accession number(s) can be found in the article/Supplementary Material.
