# Supplementary material for: A Nutrient-Based Cellular Model to Characterize Acetylation-Dependent Protein-Protein Interactions
Source: Front Mol Biosci. 2022 Mar 23;9:831758. doi: 10.3389/fmolb.2022.831758 (PMC8984119; doi:10.3389/fmolb.2022.831758)
Supplement: Supplementary file 3 [file DataSheet1.DOCX]

Supplementary Material

# Supplementary Data

**Supplementary Table S1: Plasmids and reagents used in this study**

**Supplementary Table S2: Complete MS datasets generated in this study**

# Supplementary Figures and Tables

## Supplementary Figures

**Supplementary Figure 1:** Chemical inhibitors of ACLY do not recapitulate its KO in HEK293 cells. Clonogenic assay of HEK293 parental cells incubated with and without NaOAc in medium containing stated the doses of SB-204990 (**A**), ETC-1002 (**B**), and NDI-091143 (**C**).
